# Post-term Birth and Developmental Coordination Disorder: A Narrative Review of Motor Impairments in Children

**DOI:** 10.7759/cureus.63211

**Published:** 2024-06-26

**Authors:** Manish Prasad Gupta, Dhiraj Gupta, Ali Usman

**Affiliations:** 1 Pediatrics, Shanghai First Maternity and Infant Hospital, Tongji University School of Medicine, Shanghai, CHN; 2 Radiation Oncology, All India Institute of Medical Sciences, Rishikesh, Rishikesh, IND; 3 General Surgery, Nishtar Medical University, Multan, PAK

**Keywords:** post-term, genetics, gestational age, developmental coordination disorder, motor impairment

## Abstract

A prevalent long-term medical condition in children that is rarely understood and acknowledged in educational contexts is developmental coordination disorder (DCD), which is one of the most prevalent conditions in school-aged children. Mild-to-severe abnormalities in muscle tone, posture, movement, and the learning of motor skills are associated with motor disorders. Early detection of developmental abnormalities in children is crucial as delayed motor milestones during infancy might indicate a delay in both physical and neurological development. To overcome the current condition of motor impairment, obstructing their risk factors is important to prevent the development of disability, which is already determined in the prenatal and perinatal period. Concerning the relationship with gestational age, the majority of the studies reported a relationship between DCD and preterm children. However, the entire range of gestational age, including post-term birth, has not been studied. The risk of developmental consequences such as cognitive impairments, major mental diseases, attention-deficit/hyperactivity disorder, autism spectrum disorder, and other behavioral and emotional problems increases in post-term birth, according to prior studies. Thus, this review aims to provide an overview of information linking post-term birth to children’s motor impairment, with a focus on DCD. A thorough systemic review was conducted on online databases, and only a few studies were found on the association with post-term children. Insufficient evidence made it necessary to examine more post-term cohorts in adolescence to fully determine the long-term health concerns and develop therapies to mitigate the detrimental effects of post-term deliveries.

## Introduction and background

Gestational age at birth is a highly influential component in birth outcomes. The extended health effects of children are connected to their gestational age at birth [1-3], and it is a critical component in the prediction of developmental coordination disorder (DCD) and the development of motor skills [4]. However, research has tended to focus more on preterm deliveries (fewer than 37 completed gestational weeks), which are associated with a high risk of developmental and health issues, including motor impairment in children, and less on identifying and managing post-term babies [5-7].

There is a clear pattern in the way children with motor impairments transition from crawling to walking, and the likelihood of repeated motor impairments in childhood increases even with a minor delay in the commencement of crawling and walking in early life. It is feasible to observe variations in the motor abilities of children with motor impairments as soon as six to eight months old when compared to children who are normally developing [8]. These motor impairments include difficulties with motor control (the ability to control independent motions and motor functions), motor coordination (the capacity to coordinate movements or motor activity), and visual-motor integration (performing visual details to perform fine motor assignments, such as drafting or drawing) [9-11].

A child’s individual development depends on their motor skills, which are also linked to a high quality of life. Significant long-term effects can result from even small anomalies in early motor development [12]. In addition to having severe and ongoing difficulties with everyday tasks [13,14], along with poor health, children with motor disabilities typically experience depression, low self-worth, and other psychological disorders [15-17].

DCD and cerebral palsy (CP) are two different but common motor abnormalities that start in early childhood [18]. The term CP refers to a broad range of gait and posture disorders that limit one’s activities. These disorders are thought to be severe forms of the motor impairment spectrum and are linked to permanent abnormalities of the developing baby or fetal brain [19]. Similar to CP, DCD is a general term for describing a diverse set of children whose motor coordination is significantly impaired, affecting daily activities or academic performance. The etiology of DCD is not fully understood [20]. Globally, the prevalence of CP is between 0.1% and 0.2%, but DCD is increasingly common and is estimated to impact 5-6% of children who are old enough to attend school [21].

The objective of this narrative review is to analyze the association between post-term birth and motor impairments in children, with a specific focus on DCD. While the impact of preterm delivery on various developmental and health outcomes has been extensively studied, the effects of post-term birth remain less explored. This review aims to fill this gap by synthesizing existing research to understand how post-term birth influences motor development and highlight the potential long-term consequences of DCD in children born post-term. By doing so, the review seeks to provide a comprehensive overview that can guide early intervention strategies and inform clinical practices to mitigate motor impairments in this population.

## Review

Methodology

For this narrative review, a comprehensive literature search was conducted to analyze the association between post-term birth and motor impairments in children, with a particular emphasis on DCD. Relevant articles were identified using keywords such as “child,” “children,” “motor development,” “motor impairment,” “developmental coordination disorder,” “gestational weeks,” “gestational age at birth,” “postmaturity,” and “postterm.” The databases PubMed and Google Scholar were utilized for this search.

The search strategy included reviewing peer-reviewed articles, systematic reviews, meta-analyses, and relevant clinical studies published in English. Inclusion criteria comprised studies that specifically examined the motor development and impairments in post-term-born children, as well as those focusing on DCD as a form of motor impairment. Studies involving other forms of motor impairments and those related to preterm or full-term children were excluded unless they provided comparative insights pertinent to the objective of this review.

After the initial search, articles were screened based on their titles and abstracts to determine their relevance. Full-text articles of potentially relevant studies were then retrieved and reviewed in detail. Data extracted from these articles included study design, sample size, gestational age at birth, types of motor impairments assessed, and key findings related to DCD and other motor development outcomes. This review synthesizes the findings from the selected studies to provide a comprehensive understanding of the impact of post-term birth on motor impairments in children.

Developmental coordination disorder characteristics and diagnosis

DCD is a chronic neurological illness that usually first manifests in childhood and can impair coordination and movement planning because of the mistransmission of brain signals to the body [21]. Although DCD symptoms appear in the early stages of development, they are sometimes not recognized until a child is in school, which results in lost opportunities for early intervention [22].

The labels developmental dyspraxia, minimal brain malfunction, perceptual-motor dysfunction, physical awkwardness, and, very often, the clumsy kid syndrome have all been used to characterize these children in the past [23,24]. These children were referred to as having DCD as a group in 1994 during an international consensus conference in London, Ontario [24]. The Diagnostic and Statistical Manual of Mental Disorders (DSM) (American Psychiatric Association, 2013) [21] and the 10th revision of the International Classification of Diseases (ICD-10) (World Health Organization, 1992) [25] have been some of the most extensively used classification systems, providing the diagnostic criteria for motor functioning issues in children and adolescents.

DSM-5 Diagnostic Criteria for Developmental Coordination Disorder

First: The individual’s coordinated motor skill development and execution are significantly below expectations when compared to their chronological age and opportunities for skill acquisition and application.

Second: The motor skills impairment in Criterion A substantially and repeatedly hinders age-appropriate daily living activities, affecting productivity in educational settings, prevocational and vocational activities, entertainment, and other activities.

Third: Symptoms must first appear in the early stages of development.

Fourth: Motor impairment cannot be more appropriately described by intellectual impairment, vision impairment, or any movement-impairing neurological disorder, such as CP.

Global prevalence and research on developmental coordination disorder

The hallmark of DCD is a considerable impairment in motor coordination, which frequently leads to enduring and substantial challenges with performing daily routine tasks requiring manual skills or balance [26-28]. Global estimates place the prevalence of DCD among children aged 5-11 years at 5-6% [29], with China having an even higher incidence of prevalence (8.3%) [30,31].

Research examining the vulnerability variables associated with DCD indicates that prenatal and perinatal characteristics may have an impact on the emergence of subsequent impairments [30]. Suboptimal brain development is found as a major risk factor for preterm newborns [32], and the vulnerability of DCD increases with shorter gestational ages [33,34]. Nearly half of preterm children (fewer than 37 weeks) have mild-to-moderate motor deficits [9]. Research shows that otherwise healthy late preterm newborns do not seem to experience significant developmental abnormalities in their cognitive, motor, behavioral, or socioemotional domains during childhood [35,36], although other research shows that late preterm and full-term children differ significantly in their neurodevelopment [37-39]. This suggests contradictions in the literature.

Effects of post-term delivery on motor development

Studies conducted recently have revealed a negative correlation between the short- and long-term health effects of post-term delivery (more than 41 weeks). At the ages of 18 and 36 months, post-term babies are more prone than full-term babies to have emotional and behavioral problems [40-42], as well as substantial negative consequences on cognitive measures [43]. Additionally, it was discovered that post-term children had a higher chance of developing autism spectrum disorder (ASD) [44-46] and attention-deficit/hyperactivity disorder (ADHD) symptoms [40].

Children born post-term have also been found to more commonly exhibit motor developmental deficits, including fine and gross motor abilities [47-49]. Nonetheless, compared to full-term children, major developmental milestones are achieved by post-term babies in their infancy [50]. As a result, the research on the relationship of subsequent development among post-term-born children is still conflicting. Clinical difficulties, considering the suggestion that an official diagnosis of DCD not be given until the child is five years old, restrict the ability to diagnose DCD early and accurately [51].

Inspired by the idea that early life and perinatal environments can have a significant impact on long-term health, a new preventative paradigm has surfaced in recent years [52]. A broad understanding of the effect of perinatal factors, especially the gestational age at birth on motor impairment, will aid in directing intervention efforts and creating successful population-based initiatives and strategies to treat this illness.

Factors affecting developmental coordination disorders

The causative factor of DCD is still not known, and there is not one factor that causes it. Nonetheless, progress has been achieved as a result of several investigations examining potential causes of DCD. A complicated chronologically and spatially organized series of events is involved in human brain growth, both morphologically and functionally. Research reveals a connection between DCD and diseases of the central nervous system (CNS) [53,54]. Prematurity is among the primary risk factors for motor impairment [4,30,55,56]. Low birth weight is also considered a potential risk factor for DCD [56,57].

**Figure 1:**
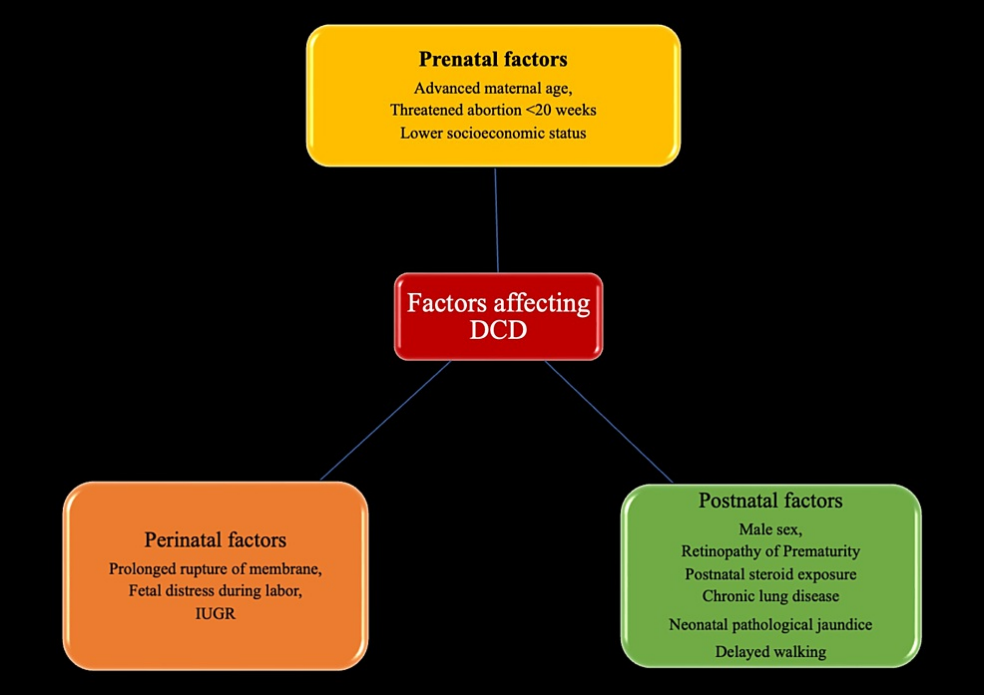
Factors affecting DCDs. The image is generated by the authors. DCD = developmental coordination disorder; IUGR = intrauterine growth retardation

Numerous investigations have examined potential variables that may be linked to DCD. Perinatal and neonatal factors associated with DCD in premature babies include prolonged rupture of membrane and retinopathy of prematurity [58], postnatal steroid exposure, male sex [59], and chronic lung disease [30]. Factors associated with DCD irrespective of gestational age include fetal distress during labor [30,60], neonatal pathological jaundice [60,61], intrauterine growth retardation, and delayed walking [33]. Prenatal factors linked to DCD include advanced maternal age [30] and threatened abortion before 20 weeks [30]. Other factors include the lower socioeconomic backgrounds of families [56]. Figure 1 shows the factors affecting DCDs.

Measurement of developmental coordination disorder

A DCD guideline was published by the European Academy of Childhood Disability in 2012, with an updated version of this guideline, known as the International Clinical Practice Recommendations for DCD, released in 2019 [51,62]. These documents provide comprehensive recommendations for the description, diagnosis, evaluation, and treatment of DCD. Although these guidelines outline general parameters for evaluating DCD outcomes, specific consensus on the most appropriate tools to employ remains limited [51,61,62]. To bridge this gap, Table 1 presents outcome measures that we suggest based on their psychometric qualities. These measures are recommended for identifying issues in school-going children with DCD, reflecting a synthesis of the guidelines and current research.

**Table 1:** Outcome measurements to identify issues in school-going children.

Diagnostic criteria	Assessment tools	Suggested measures	Key information
A	Motor functioning	1. Movement Assessment Battery for Children, 2nd edition (MABC-2). 2. Bruininks-Oseretsky Test of Motor Proficiency, 2nd edition (BOT-2)	1. Age range: 3 to 16 years 11 months; subdivisions: manual dexterity, ball skills, and balance (static and dynamic) 2. Age range: 4 to 21 years; subdivisions: running ability, agility, balance, bilateral coordination, upper limb speed, dexterity, and visual motor control
B	Activities of daily living	1. Developmental Coordination Disorder Questionnaire (DCDQ’07). 2. Movement Assessment Battery for Children Checklist, 2nd edition (MABC-2 Checklist)	1. Age range: 5 to 15 years; subdivisions: control during movement, fine motor skills and handwriting, and general coordination 2. Age range: 5 to 12 years; subdivisions: movement in a static environment, movement in a dynamic environment, and non-motor factors
C	Early onset	Parent interviews and/or tools such as the Listening for Developmental Coordination Disorder Checklist or clinical interview guidelines may be used	Developmental history as part of occupational therapy and/or physician assessment; a history of motor learning challenges should be evident since early in life
D	1. Medical examination 2. Cognitive functioning	1. Neurological exam and other tests, as required 2. Intelligence quotient testing	1. Consult a doctor to rule out any further medical or neurological causes of motor issues 2. Not required if no history of challenges with school functioning/academic achievement

The most popular tool for assessing the effectiveness of rehabilitation for individuals with DCD is the Movement Assessment Battery for Children-2 test. However, the DCD Daily and the Canadian Occupational Performance Measure (COPM) also appear to be very responsive [63]. The COPM, in particular, has been helpful in formulating rehabilitation goals [62].

Association of post-term birth with children’s motor impairment

Premature delivery raises the possibility of both immediate and long-lasting consequences, including low birth weight, newborn death, breathing issues, CP, and impairments to cognitive and motor function [2,64-65]. However, some post-term babies also experience fetal development limitations, which are most likely brought on due to poor function of the placenta that is not able to supply enough nourishment [66,67], raising the incidence of newborn encephalopathy and mortality in the first year of life [68,69]. There is uncertainty about the long-term effects.

Infants delivered beyond 41 gestational weeks are found to have high chances of developing DCD, which may result from complex post-term delivery situations such as shoulder dystocia, cephalopelvic disproportion, and prolonged labor [44]. These delivery situations raise the possibility of perinatal oxygen deficit, as previous research indicated a link between DCD and prenatal oxygen deficiency [30]. Furthermore, a post-term placenta may provide fewer nutrients and oxygen to a fetus bigger than average size, the risk of which is increased in the case of post-term delivery [70]. The post-term placenta might be connected to abnormal motor development. Despite some debate regarding the variability of motor performance in the literature [71], in contrast to atypical motor development, which is characterized by restrictions on variation and variability, typical motor development is marked by variation and the emergence of adaptive variability [72]. As a result, in post-term delivery, as children get older, it might become evident how children with typical and atypical development differ in their motor function. Identifying children who are susceptible to having DCD later in life may provide a substantial opportunity for early intervention [22].

Assessment of the impacts on children delivered after the full-term period (41 weeks and later) has received relatively less attention. Moreover, the limited amount of research conducted on the development of infants delivered after full term has yielded inconsistent results [73,74]. Among the limited studies conducted, it was discovered that at the age of two years, post-term-born infants and controls did not vary in terms of general intelligence, physical milestones, or diseases [75]. Many developmental abnormalities have been linked to prolonged pregnancy. Severe morbidity and sleep abnormalities in infancy, late societal growth and reading difficulties at one year of age, neurologic impairments, and CP have all been reported to be associated with prolonged pregnancy [76,77]. A recent study used referral to a neurologist or psychologist as a predictor of developmental issues and found that 13% of post-term children had a neurological or developmental problem at age five [78]. Studies conducted recently have revealed a negative correlation between a child’s immediate long-lasting health consequences and post-term delivery (beyond 41 weeks) [40-44].

Studies on developmental coordination disorder and post-term birth

After searching for the association of post-term birth with DCD, only a few studies on post-term-born children have been found with mixed results regarding their association with DCD. Two studies reported non-significant correlations [4,33], and one study reported a positive association [79]. According to one study, there is a higher risk of DCD screening positive for children delivered before 40 weeks of gestation, and this risk is correlated with each week of reduced gestational age. It also stated that boys are affected by DCD more than girls, and children with intrauterine growth restriction were more prone to DCD than those without it [4]. A similar negative association was reported in another study that proposed population-based and prospective design research in 2013, concluding that the risk of DCD increases with decreasing gestational age and that infants born preterm had considerably lower DCD scores than those born term and post-term [33].

Contradictory findings

However, contrary to the above studies, Hua et al. conducted a study in China in 2021 involving 152,433 children aged three to five. They found that compared to full-term children, children who were born very preterm, moderately preterm, late preterm, early term, and post-term had a higher likelihood of being placed in the suspected DCD group [79]. This finding by Hua et al. was not observed in the Danish population of roughly 23,000 to 33,000 children at seven years of age [4,33]. The reasons behind these contradictory findings could be numerous, such as the size of the samples, age at follow-up, DCD evaluation measures, and/or confounding control [80].

Developmental issues over time

While evaluating different outcomes in the study, Field et al. in 1977 found that post-term and postmature babies had different developmental problems at different periods. At delivery, poorer motor and Brazelton interaction scores, as well as more perinatal issues, were seen in the post-term and postmature newborns. Their mental and physical scores on the Denver Developmental Scale were lower at four and eight months, respectively, while their Bayley motor scores were on par with the control group [81]. Another study by van Batenburg-Eddes et al. in 2008 revealed the rate of neuromaturation lowered in infants born at 35 weeks or less and in babies born at 41 weeks or more [81]. Moster et al. in 2010 proposed population-based follow-up research using the Norwegian Medical Birth Registry on babies born between 37 and 44 weeks gestation without congenital defects and found that births at 37, 38, or 42 weeks of gestation or later were associated with a higher risk of CP compared to births at 40 weeks gestation [82]. Table 2 shows a summary of studies showing evidence of the association of post-term with children’s motor impairment.

**Table 2:** Summary of studies showing evidence of association of post-term with children’s motor impairment. CP = cerebral palsy; ICD = International Classification of Diseases; MACS = Manual Ability Classification System; GMFC = Gross Motor Function Classification System; DCD = developmental coordination disorder; DCDQ = Developmental Coordination Disorder Questionnaire; COPM = Canadian Occupational Performance Measure; ASD = autism spectrum disorder; ADHD = attention-deficit hyperactivity disorder; CNS = central nervous system; ROP = retinopathy of prematurity; IQ = intelligence quotient; OT = occupational therapy; LDCDQ = Little Developmental Coordination Disorder Questionnaire

Authors (year)	Study population	Assessment variables	Final outcome	Evidence of association
Field et al. [77]	40 post-term-born infants/40 born at term (control)	1. Brazelton Neonatal Behavioral Assessment Scale (assessing interaction and motor function)	Post-term-born children received lower Brazelton interaction and motor scores at birth and scored lower on the Denver Developmental Scale at four months	Positive
van Batenburg-Eddes et al. [81]	3,224 infants (1,576 males and 1,648 females) at corrected ages between 9 and 15 weeks/children born at 40–41 weeks of gestation (control)	Neuromotor development assessment using Touwen’s Neurodevelopmental Examination	The risk of non-optimal neuromotor development was significantly higher in infants born after 41 weeks of gestation, and the risk was higher for male and non-Dutch children	Positive
Moster et al. [82]	1,682,441 singleton-born children/children born at 40 weeks of gestation (control)	Assessment of CP based on physician diagnosis, with diagnoses registered according to ICD-99 or ICD-10	Compared with delivery at 40 weeks of gestation, delivery at 42 weeks or later was associated with an increased risk of CP for children surviving to at least four years of age	Positive
Abd Elmagid et al. [49]	1,000 children from neurology outpatient clinics	CP and motor impairments were determined through caregiver interviews, review of medical records, and direct physical examination	Preterm and post-term showed a significant association	Positive
Evensen et al. [48]	Children (n = 2,495) diagnosed with CP born between 1996 and 2015	The MACS and the GMFC were used to classify gross and fine motor functions	Term/post-term-born children are most commonly associated with CP	Positive
Rolschau et al. [83]	57,884 singleton infants born alive at weeks 39–45 of gestation	Data from the interviews and the seven-year questionnaire were linked to data from the Danish National Patient Register	No statistically significant increased risk of physical disabilities, mental disabilities, and epilepsy among children born post-term but there was an excess risk of neurological disabilities as followed for up to seven years of age	Negative
Zhu et al. [4]	22,898 singletons born between February 2007 and March 2009/children born at 40 weeks of gestation	Total score from the DCDQ	No significant increased risk of DCD was seen among children born post-term. When compared to girls, boys showed more association	Negative
Faebo Larsen et al. [33]	The study population consisted of 17,065 males and 16,289 females	Total DCD score using the validated DCDQ	The risk of DCD increases with decreasing gestational age with no significant association with post-term-born children as well, and females have a lower risk of DCD than males	Negative
Hua et al. [47]	A total of 152,433 children aged three to five years/full-term born children (39–40 weeks of gestation as reference gestation)	Outcome assessment using the LDCDQ, completed by their parents.	Every degree of prematurity at birth, early-term birth, and post-term birth was associated with suspected DCD	Positive

The finding of the above study was supported by two other studies: one reported a significant association of CP among preterm and post-term born children who were subsequently recruited from the neurology outpatient clinics over two years [49], and another study reported dyskinetic CP was more frequently linked to term/post-term delivery [83]. According to the findings of the 18-month interview and the seven-year questionnaire, there were no statistically noteworthy variations in the likelihood of physical deficits or seizures between the post-term-born children and the full-term-born children in the study that calculated the relationship between the probability of disabilities, mental impairments, and seizures throughout the initial seven years of life and post-term birth [83]. Furthermore, no statistically significant differences were discovered for CP, mental diseases, or epilepsy using data from the Danish National Patient Registry; however, the post-term group experienced more febrile seizures [83]. The various studies on post-term-born children and their association with children’s motor impairment are illustrated in Table 2.

## Conclusions

As delayed motor milestones during infancy may signal an interruption in both physical and neurological development, developmental problems in infants must be identified earlier, which allows for opportunities for early intervention. Moreover, preventing and managing post-term pregnancies should be considered due to greater perinatal mortality and morbidity. This review found mixed results, with comparatively more studies stating that children who are born after full term are more prone to experience motor impairment and developmental vulnerabilities. Further, evidence of displaying other morbidities such as sleep disorders, delayed social development, reading difficulties, neurologic handicaps, CP, ASD, and ADHD was evident in post-term-born children. Therefore, we speculate that determining these contributory factors may facilitate the identification of pregnant women at high risk and the creation of customized strategies aimed at lowering the incidence of preterm births. This might also function as a reminder to parents, teachers, and medical professionals of the need to identify and address post-term birth predictions to reduce the negative effects on the children’s health. The outcomes of this review can help doctors decide the best timing to deliver a full-term pregnancy. Post-term newborns require closer surveillance than full-term babies, even though the absolute risks of post-term birth are lower than those of preterm birth. This can be accomplished through long-term follow-up evaluations. To fully determine the long-term health hazards associated with prolonged gestation, more research on post-term cohorts in adulthood is needed, as we found limited evidence of a relationship between DCD and post-term-born children.
